# Aneurism

**Published:** 1829

**Authors:** 


31. *Aneurism. Tying the artery on the distal side.* Mr. Wardrop. The Medico-Chirurgical Review, just received, has brought us London professional news down to July. In looking through this late number, we do not discover a great deal of deep or immediate interest. The third article is on a work, by Mr. James Wardrop Surgeon, entitled 'Aneurism, and its cure by a New Operation.' We shall give a few extracts from the review. The operation, however, although denominated new, has been practised before, but not with success; and Mr. W. is fairly entitled to the praise of having shown its utility, as far at least as several cases can go.

In the first he operated himself under the most unfavorable circumstances, the patient being 75 years of age, and applied the ligature on the distal side of an aneurism of the carotid artery with perfect success. An account of three similar cases follows, the first two of which were not so successful, but the last, which was performed by Dr. Bush, is said to have been completely successful. Having thus detailed the particulars of these operations, the author proceeds to make an application of his principles to the cure of aneurism of the arteria innominata. In doing so he observes, that the process employed by nature in the spontaneous cure is precisely analogous to that which takes place in the cure by the Hunterian operation; for in both cases the coagulation of the blood proceeds from the circumference of the sac towards the centre. From this fact Mr. Wardrop was led to infer that "in cases of aneurism of the arteria innominata, the progress of the disease might be arrested by tying its two great branches, the carotid and subclavian; and although a certain portion of blood would still continue to pass along the innominata to those branches of the subclavian on the cardiac side of ligature, the ligature being necessarily placed on the subclavian artery after it emerges from between the scaleni muscles, yet such would be the diminution of the impetus of the blood in the sac, that the process of thickening of its parieties would not only go on, and thus its future increase be prevented, but even a permanent obliteration of the aneurismal cavity would be accomplished. This view is supported by the fact, that, in all cases of aneurism of any growth, we invariably find laminæ of coagula adhering to the internal surface of the sac. It is the process which nature adopts to cure the disease, by gradually filling the sac with successive deposits, until at last the cavity be so completely plugged up as no longer to admit a column of blood to pass through it. Now if we can diminish the column of blood passing through the sac of an aneurism, we most powerfully assist Nature in her efforts to consolidate the contents; for, according as the blood admitted through the sac is lessened in quantity, the greater will be the disposition in the blood contained in it to coagulate. To this some have objected that the collateral branches enlarge so much, that the usual quantity of fluid circulates through the sac. Here, again, past observation has been deficient; for, as Mr. Wardrop observes, though the collateral vessels do enlarge most considerably, immediately after the application of the ligature, yet in a very short time they again diminish in volume, though not quite to their original diameters.

These views, founded on the pathology of the disease, and the observation of the phenomena of the spontaneous cure, emboldened the author to put his plan into execution in a well-marked case of aneurism of the arteria innominata. In this case the pulsation of the right carotid, with its several branches, was not perceptible, so that its obliteration was presumed. A ligature was therefore applied to the right subclavian, and though many untoward derangements of general health retarded its progress, the cure is said to be complete; for, at the last report, the only appreciable remains of the aneurismal tumour, which originally was perceptible at the root of the neck, was an unnatural feeling of hardness above the clavicle. In this case, nature had considerably advanced in the spontaneous cure by the obliteration of the canal of the carotid, so that art was only required to assist by obliterating that of the subclavian. In those instances, however, of aneurism of the arteria innominata, where both the carotid and subclavian are quite pervious, Mr. Wardop advises the application of the ligature to the carotid artery first, and to the subclavion at a subsequent period. His view on this subject seems to have been attended with a more favorable result than could possibly have been anticipated, for, in a case of aneurism of the arteria innominata, lately operated on by Mr. Evans, of Belper in Derbyshire, the ligature of the carotid was alone sufficient for the cure, the subclavian having become *spontaneously* obliterated eight or ten days after the operation.—pp. 47–48.

Our readers will recollect the thrilling interest, inspired a few years ago, by the account of Professor Mott's operation on the arteria innominata, in a case of aneurism. The patient lived so long afterwards, as to demonstrate that the operation is not necessarily fatal, at least in the hands of that most adroit and intrepid surgeon; but there is a fearfulness, in the very idea of opening the chest, and placing a ligature around one of its great vessels, in the neighbourhood of the heart, that must and ought for ever deter all ordinary surgeons from attempting it. We cannot, therefore, but regard the results of Mr. Wardrop's experiments with the greatest satisfaction; and take pleasure in making them more extensively known, in a region of country, which has many practitioners who could successfully tie up the carotid and subclavian arteries, but would shrink from the touch of their common trunk.

